# Crossed-Brain Representation of Verbal and Nonverbal Functions

**DOI:** 10.1155/2015/301297

**Published:** 2015-01-31

**Authors:** Esmeralda Matute, Alfredo Ardila, Monica Rosselli, Jahaziel Molina Del Rio, Ramiro López Elizalde, Manuel López, Angel Ontiveros

**Affiliations:** ^1^Instituto de Neurociencias, Guadalajara, JAL, Mexico; ^2^Florida International University, Miami, FL, USA; ^3^Florida Atlantic University, Davie, FL, USA; ^4^Hospital Civil de Guadalajara Juan I. Menchaca, Guadalajara, JAL, Mexico

## Abstract

A 74-year-old, left-handed man presented with a rapidly evolving loss of strength in his right leg associated with difficulty in walking. MR images disclosed an extensive left hemisphere tumor. A neuropsychological examination revealed that language was broadly normal but that the patient presented with severe nonlinguistic abnormalities, including hemineglect (both somatic and spatial), constructional defects, and general spatial disturbances; symptoms were usually associated with right hemisphere pathologies. No ideomotor apraxia was found. The implications of crossed-brain representations of verbal and nonverbal functions are analyzed.

## 1. Introduction

For most individuals, both right- and left-handers, a language impairment (aphasia) results after left hemisphere lesions, whereas constructional and visuospatial impairments are more often observed after right hemisphere insults [1]. However, at present, right hemisphere participation for language is also recognized [2] and spatial processing is considered to be less lateralized than what was originally thought [3, 4].

Sporadically, however, language impairment is associated with right hemisphere insults [5]. Aphasia associated with right hemisphere damage in dextrals is known as “crossed aphasia” and was initially described by Bramwell in 1899 [6]. Indeed, Bramwell applied this term to two different conditions: (a) right hemiplegia and aphasia in a left-hander and (b) left hemiplegia and aphasia in a right-handed individual. Hécaen and Albert [7] suggested that the term “crossed aphasia” should be used only to refer to aphasia following right hemisphere pathology in a right-handed person, and this is how the term is currently used [8]. These data suggest that, while uncommon, there are individuals in typical populations that display a right hemisphere language dominance that has also been corroborated by fMRI studies [9] or by fMRI and the Wada test in epileptic patients [10].

The incidence of crossed aphasia is very low [11]. Hécaen et al. [12] estimated an incidence of 0.38%, while Benson and Geschwind [13] proposed a figure of approximately 1%. In large clinical samples, it has been found to be around 4% in the acute stage and 1% in the chronic stage [14]. Though it is generally accepted that crossed aphasia represents no more than 3% of all cases of aphasia [15], some authors have suggested that the incidence could be even lower [16, 17].

Disturbances usually found in cases of right hemisphere lesions, such as visuospatial defects, but associated with left hemisphere lesions in* right-handers* [18] have also been reported [19–22]. Marchetti et al. [23], for example, described a patient with a left thalamic lesion who showed motor impersistence, visuospatial dysfunction, and poor comprehension of facial expressions.

Although very early publications reflected an interest in differentiating visuoconstructive deficits associated with right or left brain damage lesions [24] or in highlighting such deficits following left brain damage [25], reports of neuropsychological correlates in nonaphasic patients following left hemisphere lesions are scarce (but see [26]). To summarize, although the literature includes several descriptions of crossed/atypical functional brain lateralization, reports on left-handed subjects are limited or unexpressed since in many series handedness is not reported.

Here, we report the case of a* left-handed* individual with a left hemisphere lesion who presented a typical “right hemisphere syndrome” (i.e., contralateral neglect, visuospatial defects, constructional difficulties, etc.). To the best of our knowledge, only two similar cases have been reported previously: Dronkers and Knight [27] analyzed a 49-year-old, left-handed woman who had suffered an infarct in the left dorsolateral prefrontal cortex that extended upwards into the inferior portions of the frontal eye fields and posteriorly into the centrum semiovale and was associated with severe hemispatial neglect, anosognosia, contralateral hypokinesia, aprosodia, and visuospatial constructive difficulties, though there was no evidence of accompanying aphasia. Also, Padovani et al. [28] studied a 54-year-old, non-right-handed man who had suffered a left hemisphere stroke associated with observations of severe right side hemineglect, transcortical motor dysprosody, spatial dysgraphia, and visuoconstructive impairments. No aphasia, alexia, right-left disorientation, or finger agnosia was noted, though a left frontotemporal subcortical lesion was documented on a CT scan.

Dronkers and Knight [27] and Padovani et al. [28] published their cases in some detail, and though there are similarities to this case, ours is unique since a more extensive assessment was performed leading to a better characterization of the patient's visuospatial dysfunction. As Dronkers and Knight suggest, this syndrome can best be explained as a reversal in hemispheric organization, since visuospatial skills are organized in the left hemisphere of left-handers, similar to the right hemisphere organization of these functions in the right-handed population. Reports of such cases allow us to better ascertain the frequency of reverse hemispheric specialization. Moreover, such unusual cases can be particularly informative in terms of attaining a better understanding of potential individual variations in the cognitive organization of the brain in relation to different variables.

## 2. Case Report

The patient is a 74-year-old, left-handed man who was a retired upholsterer with just four years of schooling. He reported that he always used his left hand for everyday tasks including writing and doing his work. Family sinistrality could not be corroborated.

He reported that about one month and a half before his current hospitalization he began to experience loss of strength in his right leg associated with difficulty in walking. Additionally, he mentioned that he had no control over his right hand and when walking in the street that hand would touch other people without him being aware of it; apparently he would make hand or foot movements without being aware of what was happening and with no control over those actions. Similarly, he sometimes lost his right shoe with no direct knowledge of any movement. One month earlier, after a fall, he had been taken to a hospital.

There, the preoperative neurological examination reported headache, disorientation, incomprehensible speech, and right hemiparesis. Three days after admission to the hospital, neurosurgery was performed. Preoperative neuropsychological evaluation could not be conducted due to his neurological status and the limited time before surgery.

**Figure 1:**
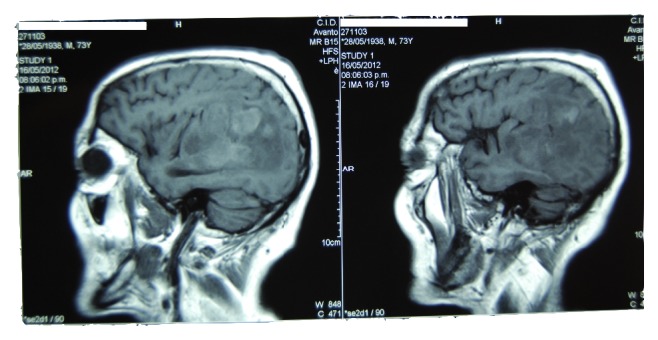
An extensive left frontal-temporal-parietal lesion is observed.

A preoperative MRI (Figure 1) revealed a lesion in the left hemisphere that involved the frontal and parietal lobes and had collapsed the ventricle. It was a hyperdense lesion with significant brain edema that suggested a space-occupying lesion. A brain tumor with a high level of malignancy that was interpreted as a probable glioblastoma multiforme was also found. The lesion was deforming the Sylvian fissure and affecting Brodmann areas 39 and 40, with an extension towards the temporal lobe.

The patient was taken to surgery to remove the tumor. A first subtotal resection (80%) of the tumor via craniostomy was performed. Two days after this surgery, neurological examination revealed Glasgow = 15, spontaneous ocular opening, isochoric pupils, photomotor and consensual reflexes, and right hemiparesis. The patient was oriented and cooperative and could follow verbal commands. However, verbal expression was altered, as his responses consisted only of signs or incomprehensible oral emissions. No complications of the surgery were reported.

A neuropsychological evaluation was performed three days after the first surgery. Although the patient was in the acute postoperative period, he was oriented with respect to time, person, and space and was pleased to be evaluated. At that time, he was unable to move his right members and his speech was hypophonic, but no language defects in phonology, lexicon, or grammar were noted. The language and speech problems that had been observed two days prior to the assessment were no longer present. Also, he was oriented, alert, and collaborative, though not particularly troubled by the difficulties he was experiencing.  Table 1 presents a summary of the tests administered and the scores achieved. “Normal” scores were considered those equal to or above the 16th percentile, percentiles 3–15 were regarded as “borderline,” and the 2nd percentile and below had been interpreted as “abnormal.”

It is evident that no language defects were observed, despite the presence of a very extensive left hemisphere tumor. Unfortunately, since there is no linguistic test for Spanish-speaking (Mexican) subjects with low schooling, no specific means of evaluating this aspect could be applied. Hence, only verbal fluency and naming were assessed through specific tests based on Mexican norms. Spontaneous speech, including dialogue with the evaluator centered on such topics as the patient's family, his life, and the reason why he was in the hospital, was possible; the patient's language was fluent, and he was able to follow both simple and complex commands.

When the patient was asked to draw a house, he narrated to the evaluator what he was drawing (e.g., a window, the garage, etc.) but the drawings were unrecognizable. His speech was clear and no phonological defects were evident. His verbal emissions were composed of simple sentences that concorded with his educational level. Semantic Verbal Fluency (animals) was below normal, but Phonological Condition (M) was normal; no verbal memory defects were found when he was asked to learn a list of 9 words in 4 trials and to recall them after 20 minutes, both spontaneously and following semantic cues. Naming in both conditions, that is, visual-verbal (confrontation naming) and verbal-verbal (finding a word when its definition is presented), was also normal. Visual-verbal confrontation naming was assessed using the Barcelona Naming test that includes 14 black-ink drawings of animals and objects, whereas verbal-verbal naming contains 6 questions, the answers to which may be an object (*what do we use to comb our hair?*), a verb (*what do we do with a pencil?*), or a place (*where do we buy medicines?*).

**Figure 2:**
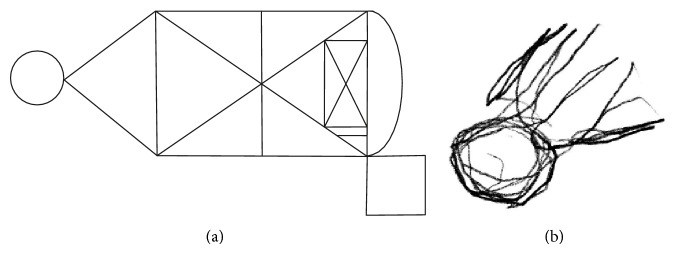
Copy of the semicomplex figure (model on the left; patient's copy on the right).

In contrast to these results, significant visuospatial and visuoconstructive impairments were clearly evident.  Figure 2 shows the copy of the semicomplex figure included in the Neuropsi: Memory and Attention test [29]. Here, significant right hemispatial neglect is evident since only the far left portion of the figure was drawn and there is iteration of several lines.

**Figure 3:**
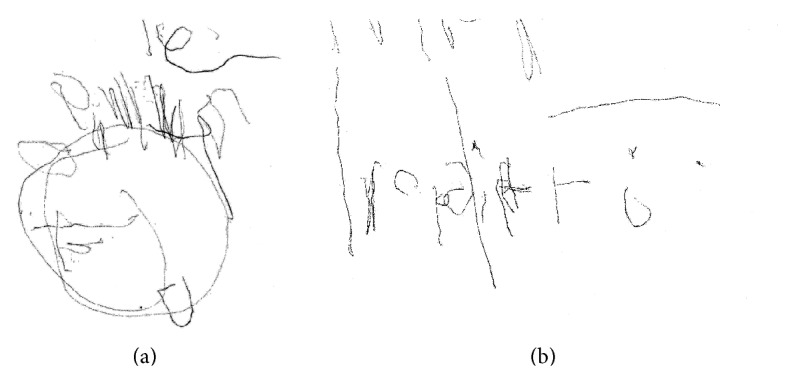
Drawings following verbal commands; a house on the right and a clock on the left.

Difficulties in drawing according to verbal commands were also observed.  Figure 3 reproduces the patient's drawings of a house and a clock. Only a few features are distinguishable, and there are one, two, three, or even more extra strokes. In fact, neither the house nor the clock is recognizable. When the patient was asked to draw a horizontal line crossing the paper from left to right, he did so only on the left side of the sheet with iterations.

**Figure 4:**
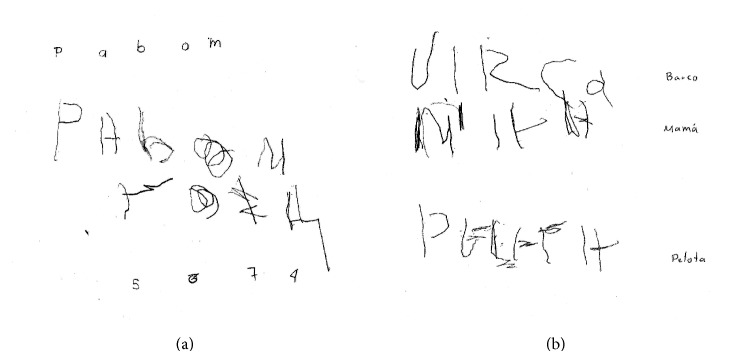
Writing by dictation:* barco* (boat),* Mama* (Mom), and* pelota* (ball) on the right; p, a, b, o, m, 5, 6, 7, 4 on the left.

Writing was also abnormal.  Figure 4 illustrates the patient's writing in a dictation exercise. Stroke iterations are observed, and some letters are poorly formed. Spatial disorganization is also noted.

**Figure 5:**
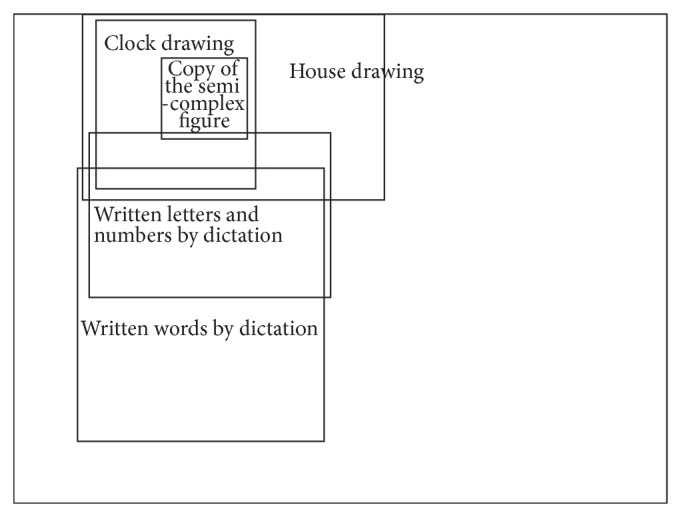
Location of examples on the sheet of paper.

The patient's drawings and strokes were performed mostly on the left side of the sheet of paper.  Figure 5 illustrates the location of the examples shown in Figures 2–4 on the sheet.

The patient could correctly recognize and read numbers, letters, and short words; however, when reading longer words and sentences, only the left side of the text presented was identifiable (e.g.,* barco* [boat] became→*ba*), while sentences were read only partially; for example, the sentence “*En el parque crecen árboles grandes*” [In the park big trees are growing] was rendered as→*En el par*, clearly suggesting right side neglect.

Interestingly, no ideomotor apraxia was seen. This disorder was tested by means of the Symbolic Gestures task, in which the patient is asked to perform different symbolic movements, such as a military salute, initially after receiving a verbal command and then by imitation, if she/he is unable to follow the verbal instruction. As the patient was able to perform this task correctly ideomotor apraxia was ruled out.

Since recall of the semicomplex figure cannot be considered a measure of memory because of the patient's severe constructional deficits and no other visual memory tests for Spanish-speaking Mexican populations with low education are available, it was not possible to perform a differential analysis of visual memory in comparison to verbal memory.

In summary, despite the extension of the left hemisphere tumor and the rapid evolution of the symptomatology, language was, broadly speaking, normal. The patient's alexia and agraphia corresponded to a (right hemisphere) spatial alexia and agraphia [31, 32]. Conversely, the patient presented with clear nonlinguistic abnormalities, including hemineglect (both somatic and spatial), constructional defects, and general spatial disturbances, all of which are usually associated with right hemisphere pathologies.

## 3. Discussion

All these data suggest that this patient presented an inverted organization of his neuropsychological functions. Despite the extension of the tumor (which involved a significant area of the left hemisphere) and its rapid evolution, no language defects were found at the time of assessment. Moreover, verbal memory was good, fluency was normal, naming was correct, grammar was correctly used, and no language understanding abnormalities were found. Also, the patient was collaborative and followed instructions easily. However, it was apparent that he was not especially concerned about his hemiplegia or his general medical condition. Difficulty in walking and alien hand (and foot) syndrome were the initial clinical manifestations of his brain tumor. The neuropsychological examination confirmed a severe visuospatial and visuoconstructive syndrome including a right neglect usually found in relation to patients with right hemisphere damage. However, no visual field was assessed to analyze a possible overlap with the neglect deficit.

This case is similar to those reported by Dronkers and Knight [27] and Padovani et al. [28]. Our patient is a left-handed individual with evident left hemisphere pathology, with no language impairment, but with very significant visuospatial and visuoconstructional defects. He kept his head tilted to the left side and, as these two authors report, a gaze deviation toward the damaged (left) hemisphere was also evident, leading to a severe neglect of objects or persons located to his right. It is especially noteworthy that no ideomotor apraxia was found. Crossed apraxia is a very unusual syndrome, but a one that has been reported occasionally (see, e.g., [33]) though studies have shown that praxis and language can be mediated by different hemispheres (see, e.g., [34]). In our case, we must assume that praxis (as well as language) was represented in the right hemisphere; that is, there was a crossed representation of both language and praxis. Unfortunately, Dronkers and Knight [27] and Padovani et al. [28] do not mention ideomotor apraxia, but this may simply suggest that praxis was normal. The fact that our patient presented with a very mild and transient language impairment leads us to suspect that language was bilateralized to some extent, a condition seen more often in left-handed subjects [35], and that the transient aphasia was in fact an expression of the acute period. Since Dronkers and Knight's [27] patient was a woman, it could be expected that both genders will present similar clinical traits.

A word must be added about this patient's writing, which was abnormal despite the absence of aphasia. Spatial agraphia is relatively rare, but when present it results from a lesion in the non-language-dominant hemisphere [7] and represents one of several features of the so-called non-dominant-hemisphere syndrome [36]. The writing traits observed in our patient were similar to those seen in patients suffering from this syndrome, that is, graphemes produced with extra strokes, writing only on the right side of the paper, blank spaces of varying size between letters, and so forth. Thus, this finding of writing impairment supports our argument that we are in the presence of a “right” hemisphere syndrome.

In the case studied by Dronkers and Knight [27], neuropsychological assessment was performed 9 days after onset, while, in the Padovani et al. [28] case, the mental status examination was carried out 2 weeks after onset. In the case we studied, in contrast, assessment took place just three days after surgery. Though it is well known that additional neurological and cognitive deficits may be present in immediate postoperative periods [37] in relation to the presence of different postsurgical phenomena, such as edema, it is important to stress that in this particular case it was not the additional deficits that made this patient so unusual (aphasia plus “right hemisphere syndrome”) but, rather, the absence of aphasia (despite the fact that he was evaluated only three days after surgery) associated with a group of deficits normally related to right hemisphere damage. It is also important to note that transient effects on cognition have been described within the first few days, or even weeks, after general surgery (i.e., non-brain procedures; see [38]). In all three cases, assessment was performed in a period ranging from 3 to 15 days after onset or surgery. Aphasia was only a transient ailment observed in our case. It is unlikely that in all three cases the “right hemisphere syndrome” could be considered as an “additional” deficit. Nonetheless, the short time interval between surgery or onset of the phenomena and neuropsychological evaluation may be judged as a limitation. To better disentangle the effects of the circumscribed lesion from those attributable to a recent surgical procedure, in future cases in which a reversed right hemisphere syndrome is suspected, a preoperative assessment followed by a postoperative one more than two weeks after surgery should be performed.

In summary, the impairment characteristics of this patient lead us to suspect the presence of a switch in hemispheric organization, since visuospatial impairments were observed after a left brain insult in this left-handed patient, together with very mild and transient language impairment. These findings suggest a reversal of hemispheric organization in this left-handed patient, since these two particular traits (normal language and impairment of visuospatial abilities) are not commonly observed in left-handers with a left hemisphere lesion. In fact, according to earlier reports, aphasia is more frequent in left-handers than right-handers after left brain damage [39]. In conclusion, it is relatively unexpected to find a left-hander with such a mild, transient language impairment after a large left hemisphere lesion. For typical populations, the study by Knecht et al. [40] demonstrates that the relationship between handedness and language is a natural phenomenon and that the incidence of right language dominance, though more frequent in left-handed than right-handed individuals, approaches 27% in these people. However, the incidence of left nonverbal function dominance in typical individuals has not yet been reported. Thus, this case could be an example of the “swapping” of functions between hemispheres.

Other limitations should also be pointed out. First, in addition to left-handedness, literacy and years of schooling are also reported to influence functional brain organization [41]. In fact, a report by Matute de Duran [42] suggests that illiterates present a lower intrahemispheric specialization for language in the left hemisphere together with a disproportionate right hemisphere involvement in language. When healthy illiterates were compared to literate individuals, lower left-side posterior parietal activation when repeating nonwords [43] and a left hemisphere attenuation of cortical event-related potentials during a verbal memory task were reported [44]. Thus, the facts that the patient in the Padovani et al. [28] paper had only 3 years of formal education and our patient left school after the fourth grade certainly attract attention. Second, our patient had a large lesion, and it is well known that identifying the eloquence of cortical areas is best conducted with smaller lesions, since it is easier to correlate cortical areas with function in those conditions. Indeed, the transient aphasia observed could be related to the fact that assessment was performed during the acute postoperative period. Third, hospital conditions did not allow us to perform any functional studies to correlate the cognitive deficits found with specific cortical or subcortical areas and thus determine the pathways that might have been affected. Fourth, strength of left-handedness was not measured, and family sinistrality was not corroborated: two issues that may well reinforce cross brain representation. Despite these limitations, clinical cases such as the one analyzed herein, together with other similar, unusual ones, can contribute to furthering our understanding of potential individual variations in the cognitive organization of the brain.

## Figures and Tables

**Table 1:** General results of neuropsychological testing.

Test	Raw score	Percentile
Neuropsi: Memory and Attention [29]		
Orientation	6	63
Digits: forwards	4	16
Digits: backwards	2	9
Serial verbal learning	4	16
Copy semicomplex figure	2.5	1
Visual detection	6	16
Successive additions	1	26
Visual tracking	1	1
Opposite reactions	1	1
Changing hand position	1	1
Semantic Verbal Fluency (animals)	7	9
Phonological verbal fluency (M)	5	16
Verbal memory: recall	2	16
Verbal memory: cued recall	3	26
Recall semicomplex figure	0	1

Barcelona test [30]		
Symbolic Gestures	10	95
Recognition: overlapped figures	8	5
Naming: visual-verbal	12	20
Naming: verbal-verbal	6	95
